# Poor cardiovascular health status among Chinese women

**DOI:** 10.1186/s12872-020-01748-y

**Published:** 2020-11-25

**Authors:** Tian-Wen Han, Yu-Qi Liu, Wei Dong, Xiao-Juan Bai, Yu-Yang Liu, Xi Su, Yu-Ming Li, Ju-Ying Qian, Mei-Xiang Xiang, Lin Cai, Qian Lin, Jing-Bo Hou, Jie Yang, Dan-Dan Li, Huan-Wan Yang, Shan-Shan Zhou, Jing Wang, Feng Tian, Xue-Qiao Zhao, Yun-Dai Chen

**Affiliations:** 1 Department of Cardiology, Chinese PLA General Hospital, Haidian District, No. 28, Fuxing Road, Beijing, 100853 China; 2 Department of Cardiology, Shengjing Hospital of China Medical University, Shenyang, 110004 China; 3 Department of Cardiology, Beijing Anzhen Hospital, Capital Medical University, Beijing, 100029 China; 4 Department of Cardiology, Wuhan Asia Heart Hospital, Wuhan, 430000 China; 5 Department of Cardiology, Logistics University of Chinese People’s Armed Police Forces, Tianjin, 300162 China; 6 Department of Cardiology, Zhongshan Hospital, Fudan University, Shanghai, 200032 China; 7 Department of Cardiology, The Second Affiliated Hospital, Zhejiang University School of Medicine, Hangzhou, 310009 Zhejiang China; 8 Department of Cardiology, The Third People’s Hospital of Chengdu, Chengdu, 610031 China; 9 Department of Cardiology, Dongfang Hospital, Beijing University of Chinese Medicine, Beijing, 100078 China; 10 Department of Cardiology, The Second Affiliated Hospital of Harbin Medical University, Harbin, 150086 China; 11 Department of Medicine, Division of Cardiology, Harborview Medical Center, University of Washington, 325 9th Ave, Box 359720, Seattle, WA GEC-3798104 USA

**Keywords:** Atherosclerotic cardiovascular disease, Cardiovascular health, Women physician, Risk factors

## Abstract

**Background:**

Systematic investigation and analysis of cardiovascular health status (CVHS) of Chinese women is rare. This study aimed to assess CVHS and atherosclerotic cardiovascular disease (ASCVD) burden in the Chinese women physicians (CWP) and community-based non-physician cohort (NPC).

**Methods:**

In this prospective, multicenter, observational study, CVHS using the American Heart Association (AHA) defined 7 metrics (such as smoking and fasting glucose) and ASCVD risk factors including hypertension, hyperlipidemia and type-2 diabetes were evaluated in CWP compared with NPC.

**Results:**

Of 5832 CWP with a mean age of 44 ± 7 years, only 1.2% achieved the ideal CVHS and 90.1% showed at least 1 of the 7 AHA CVHS metrics at a poor level. Total CVHS score was significantly decreased and ASCVD risk burden was increased in postmenopausal subjects in CWP although ideal CVHS was not significantly influenced by menopause. Compared to 2596 NPC, fewer CWP had ≥ 2 risk factors (8% vs. 27%, *P* < 0.001); CWP scored significantly higher on healthy factors, a composite of total cholesterol, blood pressure, fasting glucose (*P* < 0.001), but, poorly on healthy behaviors (*P* < 0.001), specifically in the physical activity component; CWP also showed significantly higher levels of awareness and rates of treatment for hypertension and hyperlipidemia, but, not for type-2 diabetes.

**Conclusion:**

Chinese women’s cardiovascular health is far from ideal and risk intervention is sub-optimal. Women physicians had lower ASCVD burden, scored higher in healthy factors, but, took part in less physical activity than the non-physician cohort. These results call for population-specific early and improved risk intervention.

## Background

Atherosclerotic cardiovascular disease (ASCVD) remains the leading cause of mortality worldwide and affects 6.6 million US women annually [1, 2]. However, a recent report showed that nearly 45% of women in the United States were unaware that ASCVD is the leading cause of death, and only 52% considered themselves at risk of myocardial infarction (MI) although most of them had ≥ 3 risk factors [3]. The awareness of ASCVD risk factors in Chinese women, where the ASCVD has substantially increased in recent years [4, 5], remains unknown.

To address the urgent need for primordial prevention of cardiovascular disease (CVD), the American Heart Association (AHA) proposed 7 health metrics, also referred to as Life’s Simple 7, including 4 health behaviors (smoking, body mass index (BMI), physical activity, and healthy diet) and 3 health factors (total cholesterol (TC), blood pressure (BP), and fasting blood glucose (FBG)) [6]. Three stages for each metric were created to reflect “ideal,” “intermediate,” and “poor” cardiovascular health status (CVHS) [6]. A number of studies [7–9] have evaluated CVHS and its association with ASCVD and a recent meta-analysis of 9 prospective cohort studies involving 12,878 participants reported that achieving the most ideal cardiovascular health metrics was associated with lower risk of all-cause mortality (RR = 0.55; 95% CI: 0.37–0.80), cardiovascular mortality (RR = 0.25; 95% CI: 0.10–0.63), CVD (RR = 0.20; 95% CI: 0.11–0.37), and stroke (RR = 0.31; 95% CI: 0.25–0.38) [10]. However, CVHS has not been evaluated in Chinese women, especially in Chinese women physicians (CWP).

With the aim for systematic investigation and analysis of cardiovascular health status of Chinese women doctors, we designed a prospective cohort study of CWP for ASCVD risk assessment and for implementation of effective risk reduction strategies to improve CVHS. In the current report, CVHS and ASCVD risk burden, particularly the modifiable risk factors, in CWP were evaluated compared to a community-based non-physician cohort (NPC). The levels of awareness for the modifiable ASCVD risk factors (hypertension, hyperlipidemia and type-2 diabetes (T2D)) and proportion of taking interventional actions in CWP and NPC were analyzed. This study may improve CWP’s attention to CVD, understand their own cardiovascular health status, and provide a basis for understanding Chinese women's CVD risk factors, incidence trends, and formulating prevention and treatment recommendations.

## Methods

### Study population

**Fig. 1:**
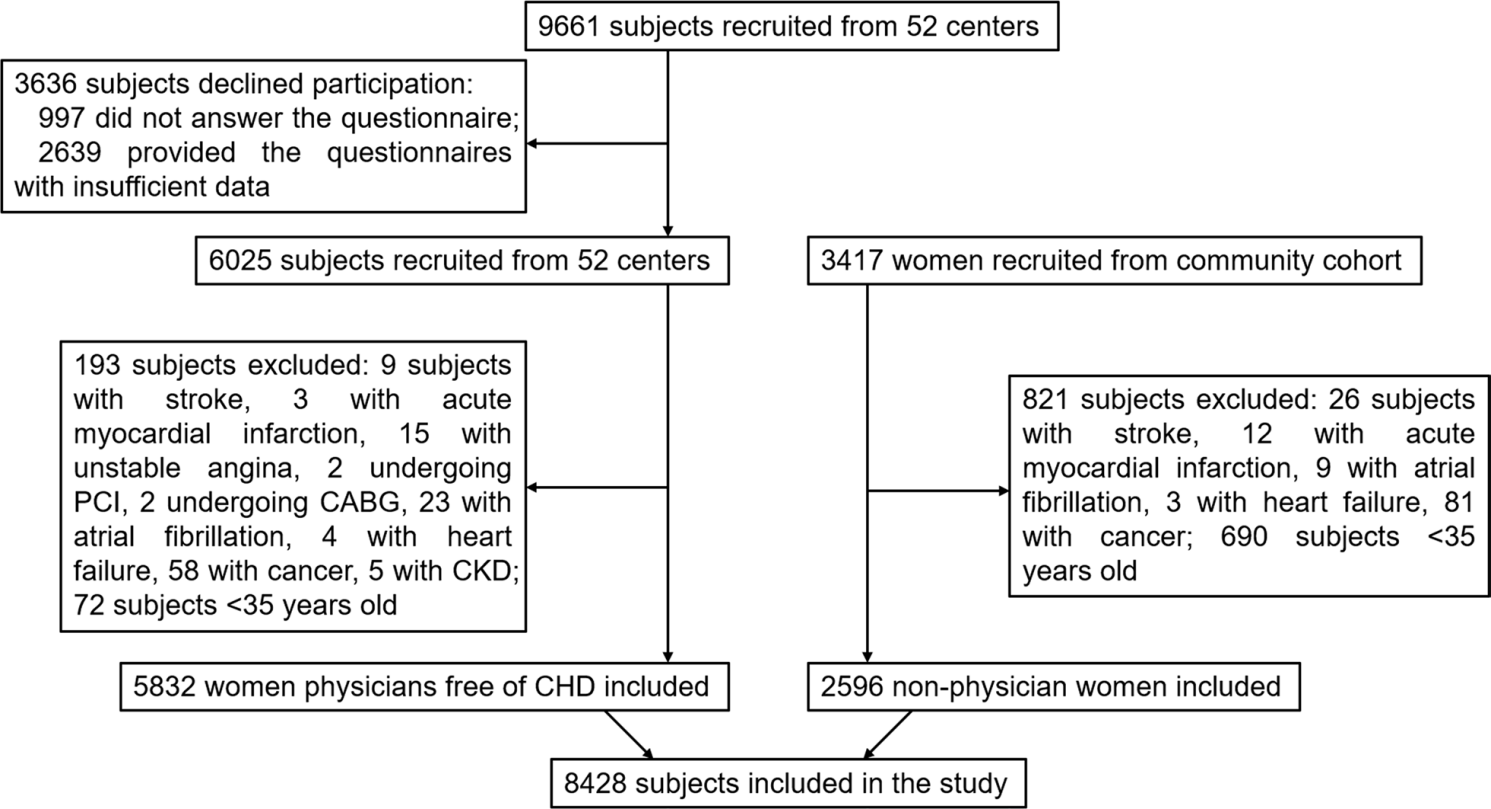
Flow chart of the study population selection

The CWP cohort study was a prospective, multicenter, observational study aimed to investigate the current CVHS and ASCVD risk factors among women physicians. The sample size of the study was initially estimated as 5740 subjects using Epi Infor Software based on the incidence of major cardiovascular events (about 1.5%) in a 4-year follow-up period in China [11]. Between January 2015 and June 2017, a total of 9661 subjects aged ≥ 35 years were recruited with a cluster sampling method across 52 study centers of 21 provinces in China (Additional file 1). An online standardized questionnaire specifically designed for this study was used (Additional file 2). Those 997 subjects who did not answer the questionnaire and 2639 subjects who provided the questionnaires with insufficient data were considered to decline research participation and had to be excluded. We received questionnaires with complete data response from 6025 subjects, which correspond to a participation rate of 62.4%. The inclusion criteria were: (1) physicians at the study centers; (2) ≥ 35 years old; (3) free of prior ASCVD, cancer and other major illness. Of the 6025 enrolled, 5832 subjects were confirmed to be free of ASCVD and other major diseases and included in the present report (Fig. 1). The study protocol was approved by the Ethics Committee of the Chinese General PLA Hospital and was in accordance with standards set forth by the Declaration of Helsinki. All participants provided their written informed consent before officially entering in the study.

To better understand the levels of ASCVD risk in CWP who have the best knowledge on CVHS relative to other population, a community-based NPC was selected from PERSUADE study [12], which is an on-going prospective cohort study in which 9078 participants were recruited from the local community hospitals geographically located in Tangshan City in northern China between January 2013 and January 2015. The inclusion criteria were: (1) women aged ≥ 35 years old; (2) providing informed consent; (3) free of prior ASCVD, cancer and other major diseases. The subjects who meet the inclusion criteria were eligible for this study. Finally, 2596 non-physician women were included in the present study (Fig. 1).

### Clinical and laboratory data collection

Participants’ demographic information, behavior characteristics (including diet, exercise, smoking status and alcohol consumption), medical history and current medications, and family history of ASCVD were collected by trained interviewers using a web-based standardized questionnaire specifically designed for this study. Body weight, height and blood pressure were obtained using the most recent annual physical examination records at each site. Laboratory measurements of lipids, FBG or glycated hemoglobin (HbA1C) were also obtained from routine annual physical examination lab reports.

### Definitions of ASCVD risk

Hypertension was defined as having any of the following 3 criteria: (1) either systolic BP (SBP) ≥ 140 mmHg or diastolic BP (DBP) ≥ 90 mmHg; (2) use of hypertension medication; (3) established diagnosis of hypertension by physicians.

T2D was defined as meeting any of the following 4 conditions: (1) FBG ≥ 7 mmol/L (126 mg/dL); (2) HbA1C ≥ 6.5%; (3) use of diabetes medication(s); (4) established diagnosis of T2D by physicians.

Hyperlipidemia was defined as having any of the following 3 measurements or 2 clinical conditions: (1) TC ≥ 5.2 mmol/L (≥ 200 mg/dL), (2) low-density lipoprotein cholesterol (LDL-C) ≥ 4.1 mmol/L (≥ 160 mg/dL), (3) triglycerides (TG) ≥ 1.7 mmol/L (≥ 150 mg/dL), (4) history of hyperlipidemia with established diagnosis by physicians; or (5) taking any lipid-lowering medication(s). The abnormal stratified standards of blood lipids for the primary prevention population of ASCVD were determined based on the 2016 Chinese guideline for the management of dyslipidemia in adults [13].

### Cardiovascular health status (CVHS) and score

CVHS was evaluated using the AHA 7 metrics including smoking, BMI, physical activity, healthy diet, TC, BP, and FBG [6]. CVHS was further classified into 3 categories: ideal, intermediate and poor.

Ideal CVHS required all 7 metrics at ideal conditions: (1) never smoked or stopped smoking for at least 12 months. (2) BMI < 24 kg/m^2^ for Chinese. (3) vigorous physical activity ≥ 75 min a week or moderate physical activity ≥ 150 min a week, or combination of both ≥ 150 min a week. Sodium and cereals intake could not be evaluated accurately due to a lack of such data collected. The ideal diet was modified in our study which included: (a) vegetables and fresh fruits ≥ 4.5 servings per day, (b) fish ≥ 3.5 oz servings per week, (c) sugar sweetened beverage consumed ≤ 450 kcal (36 oz or 4 glasses) per week, (d) meat consumed none or ≤ 2 servings per week. (5) untreated TC < 5.20 mmol/L (< 200 mg/dL). (6) untreated BP < 120/80 mmHg. (7) untreated FBG < 5.6 mmol/L (< 100 mg/dL).

Intermediate CVHS was defined as presence of any one of the 7 metrics at intermediate score and no others at poor. The criteria of intermediate CVHS included: (1) former smokers who quit less than 12 months. (2) BMI 24–27.9 kg/m^2^. (3) vigorous physical activity 1–74 min a week or moderate physical activity 1–149 min a week, or combination of both 1–149 min a week. (4) 2–3 ideal diet components. (5) TC 5.2–6.2 mmol/L (200–239 mg/dL) or treated to goal. (6) SBP 120–139 mmHg or DBP 80–89 mmHg or treated to goal. (7) FBG 5.6–7.0 mmol/L (100–125 mg/dL) or treated to goal.

Poor CVHS was defined as any of these metrics at poor levels, which included: (1) current smokers, (2) BMI ≥ 28 kg/m^2^, (3) no physical activity, (4) 0–1 diet components, (5) TC ≥ 6.20 mmol/L (≥ 240 mg/dL), (6) SBP ≥ 140 or DBP ≥ 90 mmHg, and (7) FBG ≥ 7 mmol/L (≥ 126 mg/dL).

In addition, a total score ranging from 0 to 14 was calculated as the sum of each individual component of the AHA 7 metrics (poor = 0 point, intermediate = 1 point, and ideal = 2 points) [14]. CVHS was evaluated using 6 available metrics in the community-based NPC because dietary information was not collected.

### Statistical analysis

Continuous variables are described as mean ± SD and compared using two-sample t-tests. Categorical variables are expressed as percentages and compared using x^2^ tests. A two-sided *P* < 0.05 was considered statistically significant. All statistical analyses were performed using SPSS 19.0 statistical software.

## Results

### Baseline characteristics in CWP and NPC

Figure 1 and Table 1 shows the characteristics of the 2 cohorts. Compared with NPC, CWP group were younger, with fewer smokers, lower prevalence of hypertension and T2D. However, hyperlipidemia was more prevalent in CWP than NPC. The percentage of CWP who developed ≥ 2 risk factors was lower than NPC. CWP also had lower BMI, TC and FBG, while TG and LDL-C in the CWP group and NPC group were not statistically different.

**Table 1:** Baseline characteristics between CWP and NPC

Basic characteristics	CWP (n = 5832)	NPC (n = 2596)	*P* value
Age, mean (SD), y	44 (7)	50 (10)	< 0.001
< 45, No. (%)	3417 (59)	915 (35)	< 0.001
45–54, No. (%)	1943 (33)	803 (30)	< 0.001
> 55, No. (%)	472 (8)	927 (35)	< 0.001
Post-menopause, No. (%)	838 (14)	1059 (40)	< 0.001
Hormone use, No. (%)	77(1.3)	12 (0.5)	< 0.001
BMI, mean (SD), kg/m^2^	22 (2.7)	24 (3.5)	< 0.001
Current smokers, No. (%)	54 (0.9)	52 (2)	< 0.001
Hypertension, No. (%)	373 (6)	819 (32)	< 0.001
Hyperlipidemia, No. (%)	2615 (45)	1060 (41)	< 0.001
Type-2 diabetes, No. (%)	170 (3)	194 (8)	< 0.001
≥ 2 risk factors^a^, No. (%)	459 (8)	702 (27)	< 0.001
TC, mean (SD), mmol/L	4.5 (1.1)	4.7 (0.9)	< 0.001
LDL-C, mean (SD), mmol/L	2.6 (0.8)	2.6 (0.6)	0.87
HDL-C, mean (SD), mmol/L	1.4 (0.5)	1.3 (0.3)	< 0.001
TG, mean (SD), mmol/L	1.4 (0.9)	1.4 (1.1)	0.34
FBG, mean (SD), mmol/L	5.1 (1.1)	5.3 (1.1)	< 0.001

BMI: body mass index; TC: total cholesterol; LDL-C: Low-density lipoprotein cholesterol; HDL-C: High-density lipoprotein cholesterol; TG: Triglycerides; FBG: Fasting blood glucose. ^a^Risk factors include: BMI ≥ 28 kg/m^2^, current smoking, hypertension, hyperlipidemia and type-2 diabetes

There were fewer post-menopausal subjects (14% vs 40%,*P*< 0.001); however, higher prevalence of the hormone replacement therapy (1.3% vs 0.5%,*P*< 0.001) in CWP than in NPC.

### Cardiovascular health status and score

Among 5832 CWP, only 71 (1.2%) participants had all 7 metrics at the ideal level, 502 (8.6%) at the intermediate level, while most subjects (90.1%) had at least one metric at the poor level. Each CVHS metric was compared separately between the 2 cohorts except for the diet (Table 2). In terms of the 4 healthy behaviors, smoking was low overall, but there were more current smokers in NPC than in CWP (2% vs. 0.9%,*P*< 0.001). Ideal BMI was present in 72% of CWP and 56% of NPC (P < 0.001). Ideal physical activity was seen significantly less frequent in CWP than in NPC (10% vs. 58%,*P*< 0.001). Furthermore, 84% of the physicians didn’t have any moderate or vigorous activity at all. Only 26% of CWP met the ideal diet definition.

**Table 2:** Distribution of individual Cardiovascular Health Metrics in CWP and NPC

Health metrics	CWP, No. (%) (n = 5832)	NPC, No. (%) (n = 2596)	*P* value
Smoking			
Ideal (never or quit > 12 months)	5778 (99.1)	2544 (98)	< 0.001
Intermediate (former ≤ 12 months)	0	0	
Poor (current smoking)	54 (0.9)	52 (2)	
Body Mass Index (kg/m^2^)			
Ideal (≤ 24)	4219 (72)	1452 (56)	< 0.001
Intermediate (24–27.9)	1363 (24)	807 (31)	
Poor (≥ 28)	250 (4)	337 (13)	
Physical activity			
Ideal	598 (10)	1499 (58)	< 0.001
Intermediate	329 (6)	195 (7)	
Poor	4905 (84)	902 (35)	
Healthy diet^a^			
Ideal (4 components)	1504 (26)	NA	NA
Intermediate (2–3 components)	2613 (45)	NA	
Poor (0–1 components)	1715 (29)	NA	
Total cholesterol (mg/dl)			
Ideal (< 200 without medication)	4552 (78)	1768 (68)	< 0.001
Intermediate (200–239 or treated to < 200)	1021 (18)	646 (25)	
Poor (≥ 240)	259 (4)	182 (7)	
Blood pressure (mmHg)			
Ideal (< 120/80, without medication)	3424 (59)	981 (38)	< 0.001
Intermediate (SBP 120–139 or DBP 80–89 or treated to < 120/80)	2299 (39)	1255 (48)	
Poor (sbp ≥ 140 or DBP ≥ 90)	109 (2)	362 (14)	
Fasting glucose (mg/dl)			
Ideal (< 100 without medication)	4585 (79)	2036 (78)	< 0.001
Intermediate (100–125 or treated to < 100)	1121 (19)	435 (17)	
Poor (≥ 126)	126 (2)	125 (5)	
Total score of six items (diet excluded), mean ± SD	9.0 ± 1.4	9.2 ± 1.9	< 0.001
Total score of healthy factors^b^, mean ± SD	5.1 ± 1.0	4.6 ± 1.1	< 0.001
Total score of healthy behaviors^c^, mean ± SD	3.9 ± 0.9	4.6 ± 1.2	< 0.001

SBP: systolic blood pressure; DBP: diastolic blood pressure

^a^Healthy Diet Score components: (1) vegetables and fresh fruits ≥ 4.5 servings per day; (2) fish ≥ 3.5 oz servings per week; (3) sugar sweetened beverage consumed ≤ 450 kcal (36 oz or 4 glasses) per week; (4) meat consumed none or ≤ 2 servings per week

^b^Healthy factors: total cholesterol, blood pressure, fasting glucose

^c^Healthy behaviors: smoking, body mass index, physical activity

As shown in Table 2, among the healthy factors, the prevalence of ideal BP and TC were higher in CWP compared to NPC (59% vs. 38%, 78% vs. 68%, separately,*P*< 0.001), and ideal FBG was same between the 2 cohorts (P = 0.84). CWP had a lower total score without diet information than NPC (9.0 ± 1.4 vs. 9.2 ± 1.9, *P* 0.001). As for healthy factors, CWP did better than NPC (5.1 ± 1.0 vs. 4.6 ± 1.1, *P* < 0.001); however, for healthy behaviors, CWP performed worse than NPC (P < 0.001), which was primarily due to poor physical activity (84% vs. 35%, *P* < 0.001).

### Influence of menopause on risk burden and CVHS in CWP

Overall, 838 (14%) subjects reported being post-menopause, 833 (14%) subjects were going through menopause, and 4161 (72%) subjects were pre-menopause. As shown in Table 3, the risk factors presented a stepwise increasing trend from pre-menopause to menopause and to post-menopause: BMI (22 ± 2.7 vs. 23 ± 2.7 vs. 23 ± 2.8 kg/m^2^, *P*< 0.001), TC (4.3 ± 1.0 vs. 4.4 ± 1.1 vs. 4.9 ± 1.2 mmol/L, *P* < 0.001), LDL-C (2.5 ± 0.8 vs. 2.6 ± 0.9 vs. 2.8 ± 0.9, mmol/L, *P *< 0.001), TG (1.4 ± 0.9 vs. 1.5 ± 1.1 vs. 1.6 ± 1.0 mmol/L, *P *< 0.001), and FBG (5.1 ± 1.0 vs. 5.2 ± 1.0 vs. 5.3 ± 1.2 mmol/L, *P*< 0.001). Similar trends were seen in the risk burden (hypertension, hyperlipidemia, T2D, and number of subjects with ≥ 2 risk factors) from pre-menopause to menopause and to post-menopause. CVHS total score showed a significant stepwise decrease from pre-menopause to post-menopause (P < 0.001). However, CVHS distribution of ideal, intermediate and poor was not significantly different among the 3 menopause groups (P = 0.66). The ideal diet was significantly more frequent in post-menopausal subjects (24% vs. 25% vs. 33%, *P*< 0.001).

**Table 3:** CHS by menopause

	Pre-menopause (n = 4161)	Menopause (n = 833)	Post-menopause (n = 838)	*P* value
Age, mean (SD), y	42 (5.0)	45 (6.1)	55 (6.6)	0.001
Hypertension, No. (%)	174 (4)	68 (8)	131 (16)	0.001
Hyperlipidemia, No. (%)	1732 (42)	391 (47)	492 (59)	< 0.001
Type-2 Diabetes, No. (%)	97 (2)	24 (3)	49 (6)	0.001
Current smoker, No. (%)	26 (0.6)	15 (1.8)	13 (1.6)	0.001
≥ 2 risk factors, No. (%)	292 (7)	79 (10)	88 (11)	0.001
CVHS, No. (%)				
Ideal	51 (1.2)	10 (1.2)	10 (1.2)	0.66
Intermediate	355 (8.5)	82 (9.8)	65 (7.8)	
Poor	3755 (90.2)	741 (89)	763 (91)	
Total score, mean (SD)	10.1 (1.5)	9.8 (1.6)	9.4 (1.7)	< 0.001
Ideal Diet, No. (%)	1020 (24)	205 (25)	279 (33)	< 0.001
Ideal physical activity, No. (%)	415 (10)	100 (12)	83 (10)	0.15
BMI, mean (SD), kg/m^2^	22 (2.7)	23 (2.7)	23 (2.8)	0.001
TC, mean (SD), mmol/L	4.3 (1.0)	4.4 (1.1)	4.9 (1.2)	0.001
TG, mean (SD), mmol/L	1.4 (0.9)	1.5 (1.0)	1.6 (1.0)	0.001
LDL-C, mean (SD), mmol/L	2.5 (0.8)	2.6 (0.9)	2.8 (0.9)	0.001
HDL-C, mean (SD), mmol/L	1.4 (0.5)	1.5 (0.5)	1.4 (0.5)	0.04
FBG, mean (SD), mmol/L	5.1 (1.0)	5.16 (1.0)	5.3 (1.2)	0.001

CVHS: cardiovascular health status; BMI: body mass index; TC: total cholesterol; LDL-C: Low-density lipoprotein cholesterol; HDL-C: High-density lipoprotein cholesterol; TG: Triglycerides; FBG: Fasting blood glucose

### Level of awareness and rate of treatment for modifiable risk factors

**Fig. 2:**
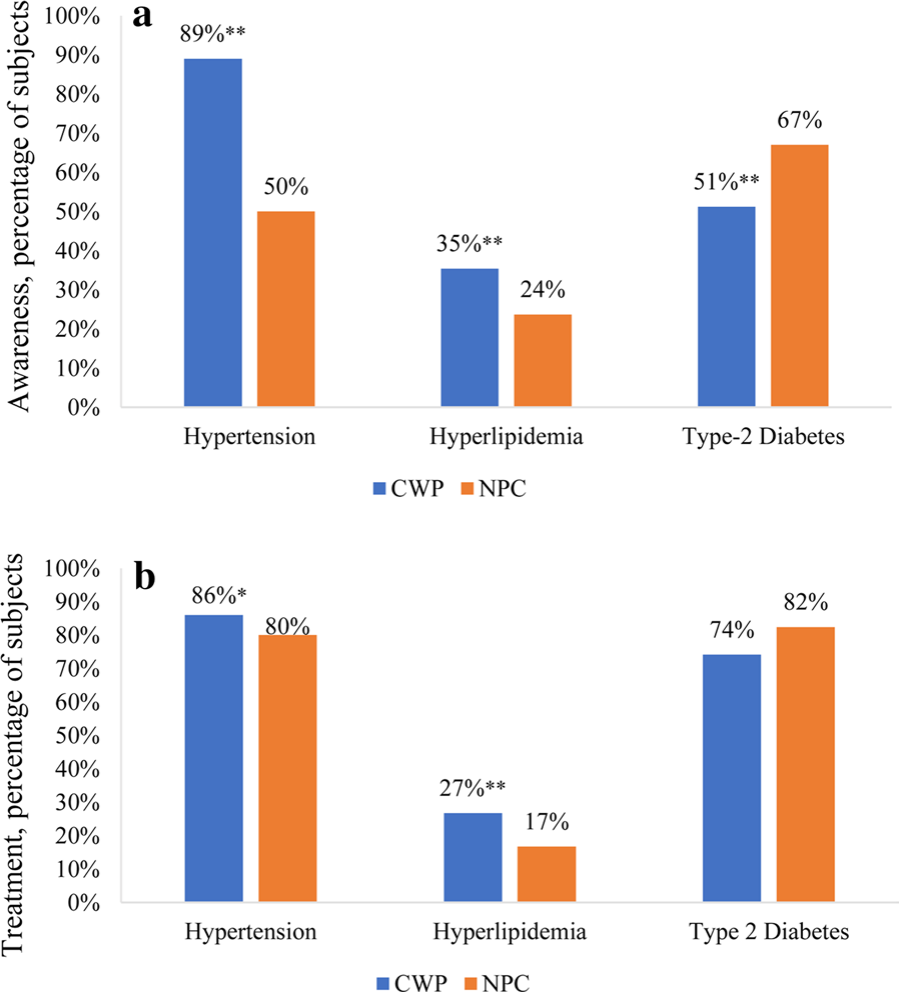
Awareness and treatment rates for three modifiable risk factors. Panel 2A describes levels of awareness. Panel 2B presents the rates of treatment for each of risk conditions. *P < 0.05, **P < 0.001

Figure 2 presents the level of awareness and rate of intervention for hypertension, hyperlipidemia and T2D in CWP and NPC. As shown in Fig. 2a, CWP group showed a significant higher level of awareness for hypertension and hyperlipidemia than NPC (89% vs. 50%, *P*< 0.001 and 35% vs. 24%, *P*< 0.001), but, a lower level for T2D (51% vs. 67%, *P*< 0.001). Among subjects who were aware of these 3 modifiable conditions, treatment rates for hypertension and hyperlipidemia were higher in CWP than that in NPC (86% vs. 80%, *P*= 0.02 and 27% vs. 17%, *P*< 0.001), but, not significantly different for T2D between the 2 cohorts (74% vs. 82%, *P*= 0.2) (Fig. 2b).

## Discussion

China is experiencing a substantial and rapid increase in ASCVD [4, 5]. Our study evaluated and compared CVHS and ASCVD risk burden in 2 populations of Chinese women, who were free from clinical ASCVD. We found that: (1) only 1.2% of CWP achieved the ideal CVHS and 90% showed at least 1 of the 7 AHA CVHS metrics at a poor level; (2) CVHS total score significantly decreased and ASCVD risk burden significantly increased in postmenopausal women although the ideal CVHS was not significantly influenced by menopause; (3) Compared with NPC, few CWP had ≥ 2 risk factors, and CWP scored significantly higher on healthy factors, a composite of TC, BP, and FBG, however, poorly on healthy behaviors, specifically in physical activity; (4) CWP also showed significantly higher levels of awareness and rates of treatment for hypertension and hyperlipidemia than NPC, but, not for T2D.

The extremely low ideal CVHS (1%) and high poor CVHS (90%) among CWP with a mean age of 44 years seen in our study are very alarming, although our observed ideal CVHS is somewhat better than that reported in previous studies [15, 16]. The younger age, medical education and health care profession in CWP may explain the differences regarding the number of subjects with ≥ 2 risk factors and CVHS healthy factor score between CWP and NPC. The results of our study complement previous findings from different populations [11, 17, 18].

It is established that healthy behaviors including non-smoking, healthy weight, diet and physical activity in CVHS are associated with lower risk of ASCVD [15, 16, 19, 20]. We found 84% of CWP with poor physical activity in our study, reporting no vigorous or moderate physical activity per week. This rate is several folds higher than the general populations in Europe (7–38%) and in the US (24% in 2005) [21, 22]. A heavy load of clinical work and physically inactive nature of the medical profession certainly add challenges for physicians to reach an ideal level of physical activity [23] By contrast, NPC in our study were more likely to be laborers as a profession which led to a lower rate (35%) of NPC with poor physical activity. Obviously, the difference between the 2 cohorts was due to professions not exercise. Nevertheless, physical inactivity among health care providers needs to be addressed for improved CVHS.

Hyperlipidemia was significantly more frequent in CWP than NPC. It is worth noting that the average level of LDL-C, 2.6 mmol/L (100 mg/dL), in our study is considered relatively normal for this population without clinical ASCVD based on current understanding. However, it is debatable whether this level of LDL-C is ideal. It has been reported that normal LDL-C levels were associated with subclinical atherosclerosis in absence of ASCVD risk factors [24]. Recently, Ference et al. [25] have suggested that plaque burden was accumulating from early age in life even with a normal LDL-C level and that ASCVD events begin to occur when LDL-C exposure threshold (Age × LDL-C mmol/L) moves beyond 129 mmol-years. To put this concept in contact with our data, mean age of 44 years and current average LDL-C level of 2.6 mmol/L in CWP; the current LDL-C exposure threshold is 113.5 mmol-years and will be higher than 129 mmol-years when CWP cohort reaches age of 50 years.

However, if the LDL-C level had been maintained at 1.8 mmol/L or lower (≤ 70 mg/dL), LDL-C exposure threshold would be 79.2 mmol-years now and would not go beyond 129 mmol-years until the cohort reaches an age of 71 years. Furthermore, previous intravascular imaging studies showed that coronary plaques may only stop progressing with the LDL-C level at ≤ 70 mg/dL [26, 27]. These data together strongly support a lower LDL-C level at a younger age.

For primary prevention of overall cardiovascular disease, AHA introduced “Life’s Simple 7”, which has been used as an effective tool for the promotion of cardiovascular disease prevention by previous studies [28, 29]. AHA definition of ideal TC is an untreated TC level less than 5.2 mmol/L (200 mg/dL) [6]. When more stringent guidelines were applied, goals of TC were even lower. The most recent consensus guidelines from the ESC/EAS established more stringent goals of ideal TC ≤ 4 mmol/L (≤ 155 mg/dl) [30]. Consistent with the 2016 Chinese guideline for the management of dyslipidemia [13] and AHA definition of ideal TC [6], our study set TC ≥ 5.2 mmol/L as one of the criteria signs for hyperlipidemia.

Another way to understand the future ASCVD risk in these 2 given populations is to project lifetime risk using the number of risk factors. Based on a published risk model in Chinese women [31], 8% and 12% cumulative ASCVD events over the next 10 years will be expected in CWP and NPC with ≥ 2 risk factors in our study.

Above and beyond aging, menopause plays an important role in increasing ASCVD risk in women [32, 33]. As shown in our study, CVHS total score decreased and ASCVD burden increased significantly during and post menopause. The ideal CVHS was not significantly influenced by menopause, because postmenopausal individuals were more conscious of healthy behaviors with better diet and maintained physical activity. A recent study has demonstrated that healthy lifestyle during menopause and midlife was associated with less subclinical atherosclerosis in women [34]. In addition, studies suggested that the follicle-stimulating hormone (FSH) rather than the estrogen may influence the 10-year ASCVD risk in post-menopausal women [35, 36].

It was estimated that promoting the overall population CVHS to ideal level will reduce deaths from ASCVD by 30% between 2010 and 2020 [37]. Our study showed that CWP had higher level of awareness and rate of treatment for hypertension than NPC and the general population [38]. The level of awareness for T2D was also higher in our study than the general population [39]. The low level of awareness and even lower rate of treatment for hyperlipidemia were consistent with those seen in the general population in China [40]. Moreover, in PERCRO-DOC survey [41], researchers found that the knowledge of current guidelines of physicians is not satisfactory and only 53.3% knew the LDL-cholesterol goal value for high-risk patients, which also reflects that physicians themselves may be not aware of the lipid profile of themselves at risk. Similarly, the awareness of CVD in public is insufficient in PERCO survey [42] in which only 43.3% knew their TC value, 30.9% knew what their target TC should be, and 53.2% of the general public have never discussed any CVD risk factor with their physicians. These results demonstrated the unmet need for ASCVD risk intervention and improved promotion of CVD prevention is warranted.

A major limitation of the present study is that the diet information was not collected in NPC, which made it impossible to fully evaluate the CVHS for NPC and to compare the health behavior between CWP and NPC. Nevertheless, our study has identified the similarities and differences in CVHS and ASCVD risk awareness and management. These findings will help to improve the precision of CVHS. Second, selection bias exists in our study such as the geographical restriction of the control cohort. Findings may not be generalizable to populations from different socio-geographic regions. In addition, cardiovascular metrics were obtained only at baseline, and changes over time were not accounted for in this study. The CWP cohort study is on-going and will evaluate ASCVD risk intervention and major events in its follow-up phase to address the importance of implementation of actions from the risk data to interventions.

## Conclusions

The cardiovascular health status is far from ideal among Chinese women. Women physicians had lower ASCVD burden, scored higher in healthy factors, but, did poorly in physical activity than the non-physician cohort. The overall ASCVD risk intervention was sub-optimal although women physicians showed higher level of awareness and rates of treatment for hypertension and hyperlipidemia. These results call for necessary population-specific early and improved risk intervention.

## Supplementary information

**Additional file 1**. The 52 participating centers of this study. 

**Additional file 2**. Questionnaire on cardiovascular risk factors of female physicians.

## Data Availability

The datasets used and/or analyzed during the current study are available from the corresponding author on reasonable request.

## Abbreviations

ASCVD: Atherosclerotic cardiovascular disease
MI: Myocardial infarction
AHA: American Heart Association
CVHS: Cardiovascular health score
BMI: Body mass index
TC: Total cholesterol
BP: Blood pressure
FBG: Fasting blood glucose
CWP: Chinese women physicians
NPC: Non-physician cohort
SBP: Systolic blood pressure
DBP: Diastolic blood pressure
T2D: Type-2 diabetes
LDL-C: Low-density lipoprotein cholesterol
TG: Triglycerides
