# Supplementary material for: Poor cardiovascular health status among Chinese women
Source: BMC Cardiovasc Disord. 2020 Nov 25;20:497. doi: 10.1186/s12872-020-01748-y (PMC7687850; doi:10.1186/s12872-020-01748-y)
Supplement: Supplementary file 1 — Additional file 1. The 52 participating centers of this study. [file 12872_2020_1748_MOESM1_ESM.docx]

Appendix- Participating Centers and PIs in the study

1. Chinese PLA General Hospital, Beijing, China.

PI: Yun-Dai Chen, email: cyundai@163.vip.com

1. Shengjing Hospital of China Medical University

PI: Xiao-Juan Bai, email: xiaojuan.bai@163.com

1. Beijing Anzhen Hospital, Capital Medical University

PI: Yu-Yang Liu, email: liuyy803803@163.com

1. Wuhan Asia Heart Hospital

PI: Xi Su, email: yaxin_suxi@163.com

1. Logistics University of Chinese People’s Armed Police Forces, Tianjin, China

PI: Yu-Ming Li, email: cardiolab@gmail.com

1. Zhongshan Hospital, Fudan University

PI: Ju-Ying Qian, email: qian.juying@zs-hospital.cn

1. The Second Affiliated Hospital, Zhejang Uninversity school of Medicine

PI: Mei-Xiang Xiang, email: xiangmxhz@163.com

1. The Third People’s Hospital of Chengdu

PI: Lin Cai, email: cailinwm@163.com

1. Dongfang Hospital, Beijing University of Chinese Medicine

PI: Qian Lin, email: linqian62@126.com

1. The second Affiliated Hospital of Harbin Medical University

PI: Jing-Bo Hou, email: jingbohou@163.com

1. Shenzhen People’s Hospital, Second Clinical Medical College of Jinan University

PI: Shao-Hong Dong, email:dsh266@medmail.com.cn

1. The First Affiliated Hospital of Chongqing Medical University

PI: Su-Xin Luo, email: [417935923@qq.com](mailto:417935923@qq.com)

1. China-Japan Union Hospital of Jilin University

PI: Ping Yang, email: [pyang@jlu.edu.cn](mailto:pyang@jlu.edu.cn)

1. The Second Affiliated Hospital of Hebei Medical Universtiy

PI: Xiu-Guang Zu, email: [zuxiuguang@medmail.com.cn](mailto:zuxiuguang@medmail.com.cn)

1. Ningbo second Hospital

PI: Hong-Hua Ye, email: Yehonghua@medmail.com.cn

1. The First Affiliated Hospital of Xinjiang Medical University

PI: Xin-Juan Xu, email: [Zcxu2002@medmail.com.cn](mailto:Zcxu2002@medmail.com.cn)

1. Xiangya Hospital of Central South University

PI: Sai-Dan Zhang, email: [zhangsaidanli@126.com](mailto:zhangsaidanli@126.com)

1. The First Affiliated Hospital of Dalian Medical University

PI: Yi-Nong Jiang, email: [yinongjiang@126.com](mailto:yinongjiang@126.com)

1. Shanxi Cardiovascular Disease Hospital

PI: Jing-Ping Wang, email: [Whw919@sina.com](mailto:Whw919@sina.com)

1. People's Hospital of Xinjiang Uygur Autonomous Region

PI: Ye-Sai Mu, email: [muyassar11@aliyun.com](mailto:muyassar11@aliyun.com)

1. Beijing Tongren Hospital, Capital Medical University

PI: Yan Fu, email: [dr_fuyan@126.com](mailto:dr_fuyan@126.com)

1. First Hospital of Jilin University

PI: Yang Zheng, email: [zhengyanghappy2005@tom.com](mailto:zhengyanghappy2005@tom.com)

1. Tianjin Chest Hospital

PI: Qin Qin, email: [qinqin6351@163.com](mailto:qinqin6351@163.com)

1. Inner Mongolia Medical University Affiliated Hospital

PI: Feng-Ying Chen, email: [fychen627@sohu.com](mailto:fychen627@sohu.com)

1. Shanghai Jiao Tong University Affiliated Sixth People's Hospital

PI: Jing-Wei Pan, email: [panjingwei@medmail.com.cn](mailto:panjingwei@medmail.com.cn)

1. The First Affiliated Hospital of Xi'an Jiao Tong University

PI: Gang Tian, email: [gangtian36@163.com](mailto:gangtian36@163.com)

1. Tianjin Medical University General Hospital

PI: Wen-Juan Zhang, email: [zwizyy2013@163.com](mailto:zwizyy2013@163.com)

1. Beijing Fuxing Hospital

PI: Ling Han, email: [hling966@sina.com](mailto:hling966@sina.com)

1. Ruijin Hospital, Shanghai Jiao Tong University School of Medicine

PI: Rong Tao, email: [rongtao@hotmail.com](mailto:rongtao@hotmail.com)

1. Shanghai Chest Hospital

PI: Xu-Min Hou, email: [xmhou@medmail.com.cn](mailto:xmhou@medmail.com.cn)

1. General Hospital of Shenyang Military Region

PI: Yaling Han, email: [hanyaling01@163.com](mailto:hanyaling01@163.com)

1. Beijing Hospital

PI: Fang Wang, email: 13911015388@163.com

1. Qilu Hospital of Shandong University

PI：Peili Bu, email: bupeili@medmail.com.cn

1. Shanghai Tenth People's Hospital, Tongji University School of Medicine

PI: Mengyun Zhu, email: [zhumy73@aliyun.com](mailto:zhumy73@aliyun.com)

1. Jiangsu People’s Hospital

PI: Qun Zhang, email: [lucyqzhang@126.com](mailto:lucyqzhang@126.com)

1. The Third Affiliated Hospital of Hebei Medical University

PI: Jun-Yan Liu, email: [Junyanliu2003@163.com](mailto:Junyanliu2003@163.com)

1. Henan Provincial People's Hospital

PI: Lixia Wang, email: [wanglixia0425@163.com](mailto:wanglixia0425@163.com)

1. Shaanxi Provincial People's Hospital

PI: Xiling Shou, email: [shouxilingsyy@126.com](mailto:shouxilingsyy@126.com)

1. The First Affiliated Hospital of Nanchang University

PI: Menghong Wang, email: [wmh666888@sina.com](mailto:wmh666888@sina.com)

1. Peking University First Hospital

PI: Meilin Liu, email: meilinliu@hotmail.com

1. Kunming General Hospital of Chengdu Military Area

PI: Lixia Yang, email: [Doctorylixia@aliyun.com](mailto:Doctorylixia@aliyun.com)

1. Guangdong General Hospital

PI: Yingling Zhou, email: [drcacocolacai@gmail.com](mailto:drcacocolacai@gmail.com)

1. Peking Union Medical College Hospital, Chinese Academy of Medical Sciences and Peking Union Medical College

PI: Shuyang Zhang, email: [shuyangzhang103@163.com](mailto:shuyangzhang103@163.com)

1. Sir Run Run Shaw Hospital, College of Medicine, Zhejiang University

PI: Guosheng Fu, email: [Fugs@medmail.com.cn](mailto:Fugs@medmail.com.cn)

1. China-Japan Friendship Hospital

PI: Jing Li, email: [nami2003@163.com](mailto:nami2003@163.com)

1. Peking University People’s Hospital

PI: Hong Chen, email: [chenhong0418@aliyun.com](mailto:chenhong0418@aliyun.com)

1. Shanghai General Hospital

PI: Shaowen Liu, email: [Shaowen.liu@hotmail.com](mailto:Shaowen.liu@hotmail.com)

1. Beijing Friendship Hospital, Capital Medical University

PI: Weiping Li, email: [xueer09@163.com](mailto:xueer09@163.com)

1. Peking University Third Hospital

PI: Lijun Guo, email: gljxwx2009@163.com

1. Peking Chaoyang Hospital, Capital Medical University

PI: Juan Zhang, email: wangzjx@263.net

1. Xijing Hospital, Fourth Military Medical University

PI: Dongdong Sun, email: [wintersun3@gmail.com](mailto:wintersun3@gmail.com)

1. Xinqiao Hospital, Third Military Medical University

PI: Bin Cui, email: cuibin_xqhospital@126.com
