# Supplementary material for: Poor cardiovascular health status among Chinese women
Source: BMC Cardiovasc Disord. 2020 Nov 25;20:497. doi: 10.1186/s12872-020-01748-y (PMC7687850; doi:10.1186/s12872-020-01748-y)
Supplement: Supplementary file 2 — Additional file 2. Questionnaire on cardiovascular risk factors of female physicians. [file 12872_2020_1748_MOESM2_ESM.docx]

Questionnaire on Cardiovascular Risk Factors of Female Physicians

This survey will take you 15-20 minutes; the health survey will involve some physical examination (biochemical) records. Please try to keep the physical examination records at hand before filling out the questionnaire. At the same time, you can prepare a measuring tape to measure waist circumference and hips.

Basic Information

1.Name:____________________________________________________

2.Address:__________

3.Post code: □□□□□□

4.Telephone:________________________ 5.Cellphone_________________

6.Email:

7.Name of your Hospital：

8.Hospital level: (1)Tertiary (level 3) (2)Secondary (level 2) (3)Primary (level 1) or community (4)others

9.Department: (1)Cardiology (2)Other department of Internal medicine (3)General (4)others

10.Technical title: (1)Senior (2)Deputy senior (3)Intermediate (4)Junior (5)others

11.Birth Date: ______/_____/_____(y/m/d)

12.Nationality: (1)Han (2)Hui (3)Man (4)Chaoxian (5)Zang (6)others

13.Education level: (1)High school (2)College (3)Master (4)Doctor

14.Marriage status: (1)unmarried (2)married (3)divorce/separated (4)widowed

15. What was your monthly household income(yuan)last year？

(1)≤2000 (2)2001-4000 (3)4001-5000 (4)≥5000

16.How often do you have a physical examination: (1)≤1year (2)1-3year (3)>3year (4)none

Lifestyle

17.Are you smoking now?

(1) Frequent smoking, smoking for ____ years, smoking ____ cigarettes a day on average (2) occasional smoking (less than one cigarette a day on average)

(3) Smoked in the past, now I have quit smoking, quit smoking for ____ years

(4) Never smoke

18. How often did you drink liquor in a year?

(1)>3 times/week (2)1-3 times/week (3)<1 time/week (4) no drink

19. How often do you drink beer in a year?

(1)>3 times/week (2)1-3 times/week (3)<1 time/week (4) no drink

20. How often do you drink wine in a year?

(1)>3 times/week (2)1-3 times/week (3)<1 time/week (4) No drink

21. What is your average daily sleep time over the past year?

(1)>8h (2)7-7.9h (3)6-6.9h (4)5-5.9h (5)<5h

Diet (The following food you consume during the past year）

22. Do you have pork?

(1) Yes, average ___ times/week or ___ times/month or____g/time (2) No

23. Do you have beef and mutton?

(1) Yes, average ___ times/week or ___ times/month or____g/time (2) No

24. Do you have chicken or duck meat?

(1) Yes, average ___ times/week or ___ times/month or____g/time (2) No

25. Do you have fish?

(1) Yes, average ___ times/week or ___ times/month or____g/time (2) No

26. Do you have eggs and other eggs?

(1) Yes, average ___ times/week or ___ times/month or____ g/time (2)No

27. Do you drink milk or yogurt?

(1) Yes, average ___ times/week or ___ times/month or____g/time (2)No

28. Do you have fruits?

(1) Yes, average ___ times/week or ___ times/month or____g/time (2)No

29. Do you have vegetables?

(1) Yes, average ___ times/week or ___ times/month or____g/time (2)No

30. Do you have legumes?

(1) Yes, average ___ times/week or ___ times/month or____g/time (2)No

32. Do you drink tea?

(1) Yes, average ___ times/week or ___ times/month or____g/time (2)No

Family History

33.Stroke: Father□ Mother□ Siblings□ None□ Unknown□

34.Acute myocardial Infarction: Father□ Mother□ Siblings□ None□ Unknown□

35.Diabetes：Father□ Mother□ Siblings□ None□ Unknown□

36.Hypertention: Father□ Mother□ Siblings□ None□ Unknown□

37.High Cholesterol：Father□ Mother□ Siblings□ None□ Unknown□

38.High Triglycerides: Father□ Mother□ Siblings□ None□ Unknown□

Personal History

39. Have you ever been hospitalized because of the following circumstances？

□stroke □Acute myocardial infarction □angina □Atrial Fibrillation

□Heart failure □Percutaneous coronary intervention □Heart bypass surgery □cancer □Kidney failure □other:_____________________(Name of diseases)

□none

40.Do you have hypertension？(1)Yes (2)No （3）Unknown

41.【If you have chosen (1) for Question 40】Hypertension for_______year？

42. 【If you have chosen (1) for Question 40】How often have you taken the antihypertensive drugs in the past year？

(1)>9 months (2)6-9 months (3)<6 months (4)Not taken

43. 【If you have chosen (1)-(3) for Question 42】Names of antihypertensive drugs you have taken in the last two weeks：(1)____________ (2)____________ (3)___________ (4)taken, but forgot the names

44.Do you have dyslipidemia？(1)yes (2)no (3)unknown{If choose (2)or(3),please turn to Question 49}

45. 【If you have chosen (1)for Question 44】Dyslipidemia for __________year？

46. 【If you have chosen (1)for Question 44】Type of dyslipidemia

(1)High LDL-C or High TC (2)High TG (3)Low HDL-C (4)unknown

47. 【If you have chosen (1)for Question 44】How long have you taken lipid-lowering drugs in the past year？

(1)>9 months (2)6-9 months (3)<6 months (4)not taken

48. 【If you have chosen (1)-(3) for Question 47】Names of lipid-lowering drugs you have taken in the last two weeks：(1)____________ (2)____________ (3)___________ (4)taken, but forgot the names

49. Do you have diabetes？(1)Yes (2)No (3)Unknown{If choose (2)or(30),please turn to Question 53}

50.【If you have chosen (1)for Question 49】Had diabetes for _______years？

51.【If you have chosen (1)for Question 49】How long have you taken hypoglycemic drugs in the past year？

(1)>9 months (2)6-9 months (3)<6 months (4)none

52. 【If you have chosen (1)-(3) for Question 51】Names of hypoglycemic drugs you have taken in the last two weeks：(1)____________ (2)____________ (3)___________(4)taken, but forgot the names

53.Have you ever taken aspirin for the past 2 weeks?

(1)Yes, to prevent the cardiovascular diseases (2)yes，but not for cardiovascular diseases (3)No

54.Do you have any physical activity during the leisure time?

(1) None (2) light physical activity (such as walking), the average number of times is ____/week or ____/month; ____ minutes/time

(3) moderate or above intensity (such as various exercises, jogging, swimming, etc.),

Average times ____/week or ____/month; ____ minutes/time

(4) Other __________________________________

Menstrual birth history

55.The age of your menarche at the age of ____year.

56.Have been pregnant for ____ times

57.Have given births for ____ times

58. [For respondents whose answer to question 56 is ≥1] Have you ever suffered from pregnancy-induced hypertension? (1) Yes (2) No (3) Have never been pregnant

59. Is your current menstrual period regular? (1) Yes, regular (2) No, irregular (3) Menopause

60. [Respondents of choice 3 for question 59] How old were you when you had a complete menopause? ______year old

61. Do you take female hormones? (1) Take it regularly (2) Take it occasionally (3) Take it often in the past (4) Don't take it

Physical Examinations

62.Height (cm)__________ 63.Weight (kg)___________

64.Waist (cm)__________ 65.Hips (cm)___________

66. The level of systolic blood pressure in the past year:（mmHg）

(1)<100 (2)100-119 (3)120-129 (4)130-139 (5)≥140 (6)Unknown

67. The level of diastolic blood pressure in the past year:（mmHg）

(1)<60 (2)60-69 (3)70-79 (4)80-89 (5)≥90 (6)unknown

Laboratory inspection: (Result of the last inspection) (If you have the result of the latest inspection, you can directly provide the laboratory test form)

68.Total cholesterol（TC）______mg/dl or______mmol/L

69.LDL-c ____mg/dl or___mmol/L

70.Triglycerides ______ mg/dl or _____mmol/L

71.HDL-C ___mg/dl or ______mmol/L

72.Fasting blood glucose _____mg/dl or _____mmol/L

73.Creatine ___mg/dl or ___mmol/L

For the cardiovascular health, What do you think is the ideal level for the following indexes?

74. SBP ______________________(mmHg) DBP_________________(mmHg)

75.Body Mass Index (BMI) ______________________

76.Waist ______________________（cm）

77.Total cholesterol（TC）（1）____mg/dl or ________mmol/L (2)unknown

78.LDL-C （1）____________________mg/dl or __________________mmol/L (2)unknown

79.Triglycerides（1）____________________mg/dl or __________________mmol/L (2)unknown

80.HDL-C （1）____________________mg/dl or __________________mmol/L (2)unknown

81.Fasting blood glucose（1）____mg/dl or _________mmol/L (2)unknown
